# Comparative analysis between digital PCR and blood culture for blood pathogen detection

**DOI:** 10.3389/fmed.2025.1615409

**Published:** 2025-09-29

**Authors:** Min Zhao, Yan Yang, Lifeng Yao, Huiming Chen, Mengqing Zhang, Yaozhen Guo, Sichao Zhang, Rong Huang, Fengrong Cai, Ling Zhang

**Affiliations:** Clinical Laboratory, Affiliated Chenggong Hospital of Xiamen University, Xiamen, China

**Keywords:** digital PCR, blood culture, pathogen, infection, sensitivity

## Abstract

**Introduction:**

Rapid and accurate identification of pathogens causing blood infections plays a pivotal role in the early diagnosis and management of infections. Digital PCR (dPCR) is a new nucleic acid amplification technology exhibiting high sensitivity for the rapid detection and absolute quantification of multiple pathogens in the blood.

**Methods:**

Herein, we conducted a retrospective study involving 149 patients with suspected infections and compared the differences between dPCR assay and blood culture in pathogen detection.

**Results:**

Blood culture showed six positive specimens and six pathogenic strains, whereas dPCR assay showed 42 positive specimens and 63 pathogenic strains. The concentrations of positive pathogens detected via dPCR varied from 25.5 to 439,900 copies/mL.

**Discussion:**

Our study demonstrated that dPCR assay has higher sensitivity, shorter detection time, and wider detection range than blood culture in blood pathogen detection, indicating its capability to support anti-infective therapy for patients.

## 1 Introduction

In recent years, the increasing incidence of infectious diseases has endangered the health of individuals and significantly affected health and socioeconomic development. When the human immune system is compromised, multiple pathogens, such as bacteria, fungi, viruses, and others, can invade the blood circulation, reproduce in the blood, and release toxins and other metabolites, causing a series of infectious symptoms. Infection with pathogens in the blood can cause various diseases or exacerbate existing conditions, with mortality rates reaching up to 50% (1). Therefore, early and accurate detection of pathogens in the blood is vital for clinical diagnosis and treatment.

Routine blood culture enables direct observation of the growth of blood pathogens and is considered to be the gold standard for pathogen detection; however, it takes a long time and has low clinical sensitivity, particularly for some pathogens that are difficult to grow in culture (2). Moreover, with the broad application of antibiotics in recent years (3), the positive rate of blood culture is greatly reduced, which makes clinical diagnosis and treatment more difficult. Therefore, the amplification of bacterial or fungal nucleic acids directly from blood samples is an effective means to identify pathogenic infections (4, 5), and thus, patients are provided with effective treatment options.

Digital PCR (dPCR) is a third-generation nucleic acid analytical technique used for the absolute quantification of nucleic acid samples, which is based on the PCR amplification of individual template molecules. This assay is not affected by complex components in the sample and can simultaneously detect multiple pathogens and resistance genes (6–8). It has the advantages of high sensitivity and quantification without relying on standard curves (9, 10). dPCR can also detect trace amounts of DNA in blood samples and identify pathogen DNA within 3–6 h, which substantially shortens the time required for pathogen detection. Several studies have reported that dPCR can be employed for the diagnosis of infectious agents, such as coronavirus, hepatitis B virus, human immunodeficiency virus, and *Mycobacterium tuberculosis* (11–14). Furthermore, dPCR has significant advantages compared with traditional PCR detection techniques, making it promising for applications in various fields, including medical diagnosis, environmental microbial detection, single-cell gene expression analysis, and quantitative analysis of transgenes (15).

This retrospective study compared dPCR and routine blood culture for the identification of multiple pathogens in blood samples. It aimed to further evaluate the application value of dPCR in the early diagnosis of blood infections and to provide more accurate information on the clinical use of antibiotics.

## 2 Materials and methods

### 2.1 Patients

This study was approved by the Ethics Committee of the Army 73rd Group Military Hospital. A total of 149 cases were retrieved from the hospital information system between January 1, 2023, and December 31, 2024, for the patients concerned. The inclusion criteria were (1) fever at admission (≥38.0°C), (2) definite focus of infection, and (3) increased white blood cell (WBC) count (≥10.0 × 10^9^/L), C-reactive protein (CRP) level (≥10 mg/L), and/or procalcitonin (PCT) level (≥0.05 ng/mL). This study retrospectively analyzed the testing records of venous blood samples maintained at our institution during 2023–2024. All samples were collected using standard aseptic procedures, and the historical records contained complete results of blood culture and dPCR.

### 2.2 Blood culture and pathogen detection

Whole blood samples were obtained for simultaneous blood culture and molecular diagnosis when symptoms of infection were clinically suspected. According to the standard clinical procedure, two sets of blood culture samples were collected for anaerobic and aerobic culture, with a venous blood collection volume of 10 mL/culture set. Then the samples were incubated at 37°C in the BacT/ALERT^®^ 3D system. After the samples tested positive, gram staining was performed, followed by a subculture on a Columbia blood agar plate at 37°C with 5% CO_2_ for 18–24 h. The infections were further detected using the Vitek 2^®^ Compact system.

### 2.3 DNA extraction and dPCR assay

Whole blood sample for dPCR analysis was obtained from each patient in a tube containing ethylenediaminetetraacetate. Then, the plasma was immediately separated via centrifugation at 1,600 × g for 10 min. Plasma DNA was extracted using nucleic acid extraction or purification kits (Pilot Gene Technology, Hangzhou, China) (16, 17) and the Auto-Pure10B Nucleic Acid Purification System according to the manufacturer’s instructions. DNA (100 μL) was collected for dPCR analysis on the same day.

To detect the pathogens, dPCR assay was performed using a droplet digital PCR system (Pilot Gene) according to the manufacturer’s instructions. Briefly, 15 μL of extracted DNA was added to dry powder containing the fluorescent probe and primer, vortexed, and then centrifuged. Subsequently, the reaction solution was separately added to each channel sample well. Droplet production was performed using the equipment, and PCR reaction was carried out according to the instructions. After the above process was completed, the cartridge was moved into the chip scanner for droplet analysis. Six fluorescence channels (FAM, VIC, ROX, CY5, CY5.5, A425) were detected to identify the microorganisms in each panel, and data were analyzed using the Gene PMS software version. According to the manufacturer’s validation, the pre-designed panel of the dPCR kit does not include primers/probes for *Salmonella enterica* or *Streptococcus sanguinis*, as these pathogens are not within the intended clinical scope of the kit.

### 2.4 Data analysis

Statistical analyses were conducted using the SPSS software version 27.0. Continuous variables that followed a normal distribution were expressed as mean ± standard deviation. Contrarily, continuous variables that did not follow a normal distribution were expressed as median and interquartile ranges (IQR). Comparisons between the groups were performed using the Mann–Whitney U test. Meanwhile, categorical variables were expressed as frequencies (percentages) and compared using the chi-squared test. The *p* value of 0.05 was considered statistically significant.

## 3 Results

### 3.1 Characteristics of the patients’ clinical conditions

This study included 149 patients. They were divided into the following groups: positive dPCR group, which consisted of 42 patients [22 (52.4%) were men], with a median age of 28.5 (IQR, 7–67) years, and negative dPCR group, which consisted of 107 patients [65 (60.7%) were men], with a median age of 6 (IQR, 3–31) years. The age difference between the groups was statistically significant (*p* < 0.001, Table 1). For inflammatory factors, the median WBC count, CRP level, and PCT level in the dPCR-positive group were 9.81 × 10^9^ (IQR, 6.03–16.78 × 10^9^)/L, 50.81 (IQR, 18.03–143.44) mg/L, and 0.39 (IQR, 0.13–1.09) ng/mL, respectively. These values showed statistical significance in relation to the value in the negative dPCR group (*p* < 0.05, Table 1). The average detection time of dPCR was 4.8 ± 1.3 h, which was significantly shorter than the average time required for blood culture (94.7 ± 23.5 h) (Supplementary Table 1).

**Table 1:** Comparative analysis of the general clinical characteristics of patients.

Clinical characteristics	Positive dPCR (*n* = 42)	Negative dPCR (*n* = 107)	Z/χ^2^	*P*
Gender				
Male (n,%)	22 (52.4)	65 (60.7)	0.869	0.351
Age (years)	28.5 (7–67)	6 (3–31)	−4.167	<0.001
WBC, median (IQR) × 10^9^/L	9.81 (6.03–16.78)	7.53 (5.66–11.66)	−1.970	0.049
PCT (ng/mL), median (IQR)	0.39 (0.13–1.09)	0.19 (0.12–0.41)	−2.203	0.028
CRP (mg/L), median (IQR)	50.81(18.03–143.44)	22.29 (4.85–84.84)	−2.447	0.014

### 3.2 Pathogens detected by blood culture

In this study, blood samples from 149 patients demonstrating symptoms of infection were tested, of which six showed positive blood culture (Table 2). Five species and six strains of pathogens were detected in the positive blood culture, including three gram-negative and two gram-positive bacteria (Supplementary Figure 1). Further breakdown of the results of the gram-negative bacteria revealed one strain of *Klebsiella pneumoniae*, one strain of *Pseudomonas aeruginosa*, and one strain of *Salmonella enterica*. The gram-positive bacteria included two strains of *Staphylococcus aureus* and one strain of *Streptococcus sanguinis*. Of these six positive samples, four blood cultures were consistent with the results of the pathogens detected by dPCR. In patients 42 and 78, the pathogens detected in the blood culture were *Streptococcus sanguinis* and *Salmonella enterica*, respectively (Table 2). Both pathogens were beyond the detection range of dPCR.

**Table 2:** Comparison between dPCR and blood culture for pathogen detection in patients with positive blood cultures.

Group	Sample ID	Blood culture results	dPCR results
Gram-negative bacteria	77	*Klebsiella pneumoniae*	*Klebsiella pneumoniae*
	87	*Pseudomonas aeruginosa*	*Pseudomonas aeruginosa*
	78	*Salmonella enterica*	*Acinetobacter baumannii*
Gram-positive bacteria	88	*Staphylococcus aureus*	*Staphylococcus aureus*
	52	*Staphylococcus aureus*	*Staphylococcus aureus*
	42	*Streptococcus sanguinis*	*–*

### 3.3 Pathogens detected by dPCR

dPCR detected 42 blood samples as positive (Table 3). These positive samples had 13 species and 63 strains of pathogens, including eight bacteria, two fungi, and three viruses (Supplementary Figure 1). Among them, the most detected bacteria were *Acinetobacter baumannii* (*n* = 11), followed by *Streptococcus* spp. (*n* = 10) (Supplementary Figure 2). *Streptococcus* spp. mainly included *S. pneumoniae*, *S. anginosus*, *S. pyogenes*, *S. mitis*, and *S. agalactiae*. For patient 78, the main pathogens were *Acinetobacter baumannii* and *Salmonella enterica*. The latter was not detected as it was beyond the detection range of dPCR. Among the detected bacteria, the strain with the lowest detected concentration was *Acinetobacter baumannii* (25.5 copies/mL in the blood), and the strain with the highest concentration was *Streptococcus* spp. (141,460 copies/mL in the blood). High levels of cytomegalovirus (439,900 copies/mL in the blood) were detected in patient 33. In the dPCR detection range, there were 14 cases of polymicrobial infections, including 10 cases of double infections, two of triple infections, one of quadruple infections, and one of quintuple infection, suggesting that dPCR assay has high capacity to detect multiple microorganisms.

**Table 3:** Pathogens detected by dPCR and blood culture within the detection range of dPCR.

Sample ID	dPCR results	Concentration in the blood (copies/mL)	Blood culture results
77	*Klebsiella pneumoniae*	13,635	*Klebsiella pneumoniae*
87	*Pseudomonas aeruginosa*	5,627	*Pseudomonas aeruginosa*
88	*Staphylococcus aureus*	2792.5	*Staphylococcus aureus*
52	*Staphylococcus aureus*	8,729	*Staphylococcus aureus*
78	*Acinetobacter baumannii*	791	*Salmonella enterica*
136	*Acinetobacter baumannii*	609	Negative
	*Enterococcus faecium*	60	
	*Pseudomonas aeruginosa*	71,091	
	*Staphylococcus aureus*	70	
	*Streptococcus* spp.	258	
40	*Acinetobacter baumannii*	76.5	Negative
16	*Acinetobacter baumannii*	78.5	Negative
09	*Escherichia coli*	52	Negative
07	*Streptococcus* spp.	127.5	Negative
68	*Acinetobacter baumannii*	52	Negative
	*Escherichia coli*	62.5	
28	*Acinetobacter baumannii*	68	Negative
	*Candida* spp.	202.5	
	*Pseudomonas aeruginosa*	4115.5	
	*Streptococcus* spp.	339	
96	Coagulase-negative *Staphylococcus*	310	Negative
	*Enterococcus faecium*	140.5	
60	*Klebsiella pneumoniae*	43.5	Negative
71	*Acinetobacter baumannii*	40.5	Negative
29	*Pseudomonas aeruginosa*	28	Negative
79	*Candida* spp.	85	Negative
92	*Candida* spp.	65	Negative
10	*Candida* spp.	210.5	Negative
15	*Pseudomonas aeruginosa*	205	Negative
89	*Streptococcus* spp.	61.5	Negative
101	*Streptococcus* spp.	1811	Negative
80	*Streptococcus* spp.	107.5	Negative
	Genus *Aspergillus*	32.5	
61	*Klebsiella pneumoniae*	27	Negative
126	*Pseudomonas aeruginosa*	46.86	Negative
	*Escherichia coli*	33.66	
66	*Acinetobacter baumannii*	31	Negative
16	*Streptococcus* spp.	291	Negative
50	*Streptococcus* spp.	141,460	Negative
	Epstein–Barr virus	2354.5	
99	*Acinetobacter baumannii*	25.5	Negative
43	*Acinetobacter baumannii*	52	Negative
	Epstein–Barr virus	88	
	*Streptococcus* spp.	122.5	
98	*Acinetobacter baumannii*	79	Negative
	*Pseudomonas aeruginosa*	326.5	
113	*Enterococcus faecium*	68.5	Negative
	Epstein–Barr virus	42.5	
	Herpes simplex virus type 1 and type 2	4,177	
01	*Enterococcus faecium*	41	Negative
	Epstein–Barr virus	100.71	
20	*Candida* spp.	133.5	Negative
37	*Candida* spp.	59.5	Negative
	*Enterococcus faecium*	30	
41	*Enterococcus faecium*	35.5	Negative
95	*Candida* spp.	66.5	Negative
67	Epstein–Barr virus	44	Negative
06	Epstein–Barr virus	9,330	Negative
33	Cytomegalovirus	125.5	Negative
	Epstein–Barr virus	439,900	
47	*Pseudomonas aeruginosa*	379.5	Negative
	*Streptococcus* spp.	54	
85	Epstein–Barr virus	32.5	Negative

### 3.4 Comparison between dPCR and blood culture

Of the 149 tested blood samples, 42 and 107 were detected as positive and negative for pathogens by dPCR, respectively, with a positivity rate of 28.2% (42/149). Six samples were found to be positive in the blood culture, with a positivity rate of 4% (6/149) (Table 4). *Streptococcus sanguinis* and *Salmonella enterica* were identified solely by blood culture was outside the dPCR detection range. Therefore, four of the six positive blood culture cases were positive by both blood culture and dPCR and had the same strain. For patient 78, the main pathogens were *Acinetobacter baumannii* and *Salmonella enterica*. Therefore, the sample size for simultaneous positive dPCR and blood culture was five. Antibiotics were used in 17 of the 42 samples that were positive in dPCR and negative in blood culture. The clinical symptoms and other indicators in six of these cases are presented in Supplementary Table 2. Moreover, 14 polymicrobial infections were detected in 42 (28.6%) positive dPCR samples, whereas polymicrobial infections were not detected in the blood culture samples (Table 3).

**Table 4:** Positive detection of the dPCR and blood culture methods.

Blood culture	dPCR		Total
	+	−	
+	5	1	6
−	37	106	143
Total	42	107	149

## 4 Discussion

Early diagnosis of pathogen infections is important for developing effective treatment regimens, controlling the progression of infection, and improving patient prognosis (18). Blood culture is considered to be the gold standard for the diagnosis of pathogens; however, it often takes 2–3 days. Furthermore, infections caused by multiple pathogens are often difficult to identify, and antibiotic therapy during this period may reduce the overall susceptibility (19). dPCR assay has significant advantages over blood culture, rapidly providing results within 3–6 h and dramatically reducing the mean diagnostic time. Previous studies have demonstrated the ability of dPCR to rapidly detect pathogens in whole blood (10, 20). In our study, dPCR detected various pathogens, including bacteria, fungi, and viruses, in either single infection or coinfections.

In this retrospective study, we made a direct comparison between dPCR and blood culture for the detection of pathogens in the blood. Of the 149 blood samples collected, 42 positive samples and 63 pathogen strains were detected by dPCR, whereas only six positive samples and six pathogen strains were detected by blood culture. The positive rate of dPCR was 28.2%, which was substantially higher than that of blood culture (4%). The sensitivity of digital PCR detection was as high as 83.33% (5/6), and the specificity was 74.13% (106/143). The reason for the relatively low specificity might be the high sensitivity of digital PCR in monitoring pathogens, as well as the potential false-negative results caused by the application of antibiotics in blood culture tests. Of the 42 patients who tested positive in dPCR, the concordance rate with the blood culture results reached as high as 66.7% (4/6). This result indicates that dPCR is superior to blood culture in terms of detection rate and shows potential for use in the rapid and early detection of pathogens. In addition, our study relied on a commercial dPCR kit with a fixed panel of targets, which may not cover rare or atypical pathogens (e.g., *Streptococcus sanguinis, Salmonella enterica*). This indicates that the detection range of dPCR is limited, and there is a certain risk of failure to detect some pathogens in the clinic. Future studies could incorporate custom primers or expanded kits for broader coverage.

The high sensitivity of dPCR ensures that pathogens can be accurately detected even at low levels, which is crucial for the timely treatment of patients with severe conditions (1, 21). Previous studies have reported that molecular tests, such as PCR, can identify positive cases in 10–40% of initially negative blood cultures, thereby improving patient prognosis through targeted antibiotic therapy (22). In our study, dPCR detected eight bacteria, two fungi, and three viruses in 37 culture-negative patients. Moreover, infectious diseases are involved in the pathogen concentrations (23, 24). dPCR has a detection limit of 0.5 copies/mL for most pathogens, except for *Streptococcus* spp., *Candida* spp., and coagulase-negative *Staphylococcus*, which have a detection limit of 1 copies/mL. In this study, dPCR detected a range of concentrations of different bacteria in the blood from 25.5 to 141,460 copies/mL, fungi from 32.5 to 210.5 copies/mL, and viruses from 32.5 to 439,900 copies/mL, which showed the higher sensitivity of dPCR. In addition, dPCR can rapidly provide accurate quantitative data of the pathogen, which can reflect the severity of infection and inflammation. Therefore, dPCR can monitor patients’ conditions in real time and provide early warning to clinicians (25).

In this study, 37 samples were detected as positive by dPCR but negative by blood culture. In the patients’ medical records, it was found that the patients generally showed symptoms such as fever, cough, and elevated WBC count, and 45.9% (17/37) of patients were treated with antibiotics before blood sampling, which may be one of the reasons for the negative blood culture. Second, dPCR quantitative validation showed that the majority of samples had pathogen loads below the blood culture detection limit (<5 CFU). Although dPCR can detect the DNA of dead microorganisms, the positive samples in this study were accompanied by corresponding clinical symptoms and elevated inflammatory markers, suggesting their clinical relevance. The dPCR method enables the detection of pathogens in the presence of nucleic acid fragments in the blood, which also provides more valuable information for the detection of unknown pathogens or their DNA. Moreover, many patients with symptoms may receive antibiotic treatment before sample collection, which may reduce microbial culture sensitivity. Contrarily, the application of antibiotic treatment had no significant effect on the sensitivity of dPCR assays.

Our study found that the levels of CRP and PCT in the dPCR positive group were significantly higher than those in the dPCR negative group (*p* < 0.05). CRP is an acute time phase protein that increases when the body undergoes inflammatory responses and tissue damages caused by infections. PCT is a specific indicator for diagnosing bacterial infections (26). Under normal conditions, CRP and PCT are expressed at extremely low levels. When pathogenic bacteria infection occurs and inflammatory response and tissue damage occur, their levels increase sharply. Relevant studies have demonstrated that as the severity of infection increases, the levels of CRP and PCT also elevate (27, 28). The results of our study also showed that the more types of pathogenic bacteria detected, the higher the pathogen load, the more severe the inflammatory response in the body, and the higher the levels of CRP and PCT. Therefore, clinicians can use indicators such as CRP and PCT to evaluate whether patients with dPCR positive results have an infection and its severity.

Herein, dPCR showed a higher detection rate for polymicrobial infections (47%) in the same sample than blood culture. This may be due to the lower detection limit of dPCR as well as the slow growth and high nutrient requirements of some pathogens during blood culture. In addition, to determining microbial infection based on pathogen concentration, clinicians also consider the results of blood biochemistry analyses, clinical presentation, and the indicators of infection or inflammation, such as CRP and PCT. The above indicators were used to further determine whether a positive dPCR result for a low pathogen concentration indicates a true infection.

In addition, a study on bloodstream infections demonstrated that the limit of detection of dPCR for *Klebsiella pneumoniae* was 0.27 copies/μL (29), and that in patients with *Staphylococcus*, a high bacterial DNA load detected in the blood by dPCR was associated with sepsis, infective endocarditis, and mortality (30). The above indicates that the quantitative results of dPCR are related to the pathogenicity and clinical outcomes of the pathogen. However, we did not assess dynamic changes in bacterial DNA load identified by dPCR detection method. Future research plans to collect continuous samples to evaluate the diagnostic value of dPCR in assessing the efficacy of antimicrobial therapy.

## 5 Conclusion

Accurate and early detection of a pathogen helps clinicians select appropriate antibiotics for the patients. This study demonstrates that dPCR can detect a variety of pathogens, including bacteria, fungi, and viruses, with higher sensitivity and faster speed of detection. In addition, the sensitivity of dPCR is not affected by the application of antibiotics, which makes dPCR more promising in clinical applications. However, the number of pathogen-positive samples collected in this study was relatively small. Therefore, we will continue to collect clinical samples in the future.

## Data Availability

The original contributions presented in the study are included in the article/Supplementary material, further inquiries can be directed to the corresponding author.
