# Supplementary material for: Comparative analysis between digital PCR and blood culture for blood pathogen detection
Source: Front Med (Lausanne). 2025 Sep 29;12:1615409. doi: 10.3389/fmed.2025.1615409 (PMC12515817; doi:10.3389/fmed.2025.1615409)
Supplement: Supplementary file 1 [file Data_Sheet_1.docx]

**Supplementary data**

**Comparative analysis between digital PCR and blood culture for blood pathogen detection**

**Supplementary Table 1.** Clinical characteristics of patients.

| Clinical characteristics | n = 149 |
| --- | --- |
| Gender |  |
| Male (n,%) | 87(58.4%) |
| Age (years) | 7(3.5–50.5) |
| WBC, median (IQR) × 10^9^ /L | 8.05 (5.76–12.32) |
| PCT (ng/mL), median (IQR) | 0.21 (0.12–0.52) |
| CRP (mg/L), median (IQR) | 36.15 (6.12–94.83) |
| Average time of dPCR detection (hour) | 4.8±1.3 |
| Average time of blood culture (hour) | 94.7±23.5 |


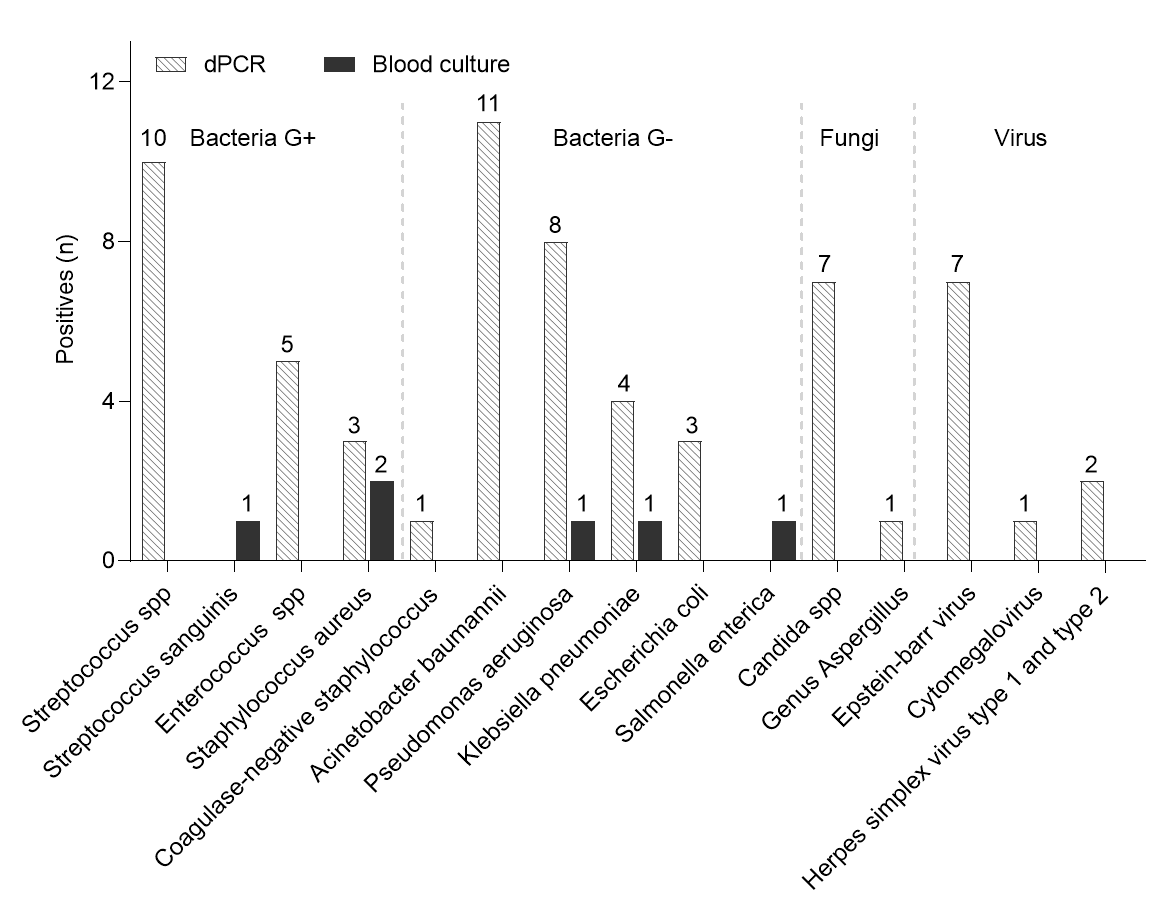


**Supplementary Fig. 1.** Distribution of the pathogens in the samples from the blood culture and dPCR assay. Bacteria G+: gram-positive bacteria. Bacteria G: gram-negative bacteria.

**
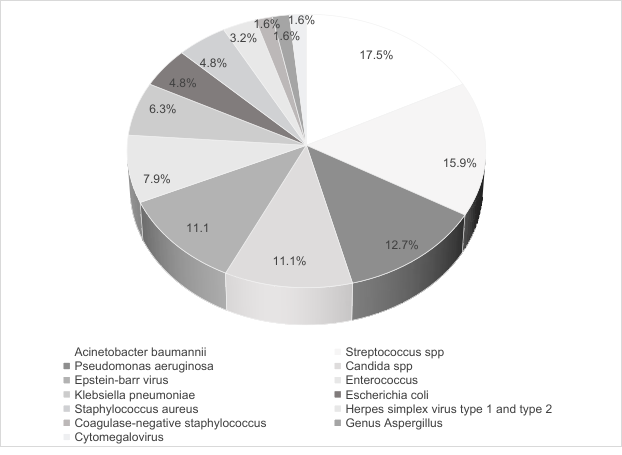
**

**Supplementary Fig. 2.** Distribution of pathogens detected by digital PCR.

**Supplementary Table 2.** Clinical signs of partial positivity for dPCR with negative blood culture.

| Sample ID | dPCR | Blood culture | Clinical symptoms |
| --- | --- | --- | --- |
| 37 | *Candida*,  *Enterococcus faecium* | Negative | Recurrent fever with cough, elevated CRP level, and coarse breath sounds in both lungs.  Antibiotic treatment was administered before blood sampling. |
| 41 | *Enterococcus faecium* | Negative | Repeated coughing and chest radiography showed increased and blurred texture in both lungs, and bilateral tonsils were enlarged to the first degree. Antibiotic treatment was administered before blood sampling. |
| 98 | *Acinetobacter baumannii* and *Pseudomonas aeruginosa* | Negative | The temperature was up to 40°C, with heat spikes 3–4 times/day. The bilateral tonsils were enlarged for a time, and respiratory sounds were coarse in both lungs. Antibiotic treatment was administered before blood sampling. |
| 99 | *Acinetobacter baumannii* | Negative | High fever with chills accompanied by significant increases in inflammation indicators (CRP: 72.55 mg/L). CT showed bilateral inflammation. Antibiotic treatment was administered before blood sampling. |
| 126 | *Pseudomonas aeruginosa*, *Escherichia coli* | Negative | Abdominal pain, diarrhea, and vomiting, accompanied by fever. Antibiotic treatment was administered before blood sampling. |
